# Development and validation of an innovative administration system to facilitate controlled holmium-166 microsphere administration during TARE

**DOI:** 10.1186/s40658-024-00692-6

**Published:** 2024-10-16

**Authors:** Meike W. M. van Wijk, Gerhard van Wolfswinkel, Mark J. Arntz, Marcel J. R. Janssen, Joey Roosen, J. Frank W. Nijsen

**Affiliations:** 1 Department of Medical Imaging, Radboud University Medical Center, Nijmegen, The Netherlands; 2 R&D TIO, Quirem Medical B.V. – A Terumo Company, Deventer, The Netherlands

**Keywords:** TARE, SIRT, Liver, Holmium, Image-guided, Personalisation, Treatment delivery

## Abstract

**Background:**

To develop and validate a novel administration device for holmium-166 transarterial radioembolisation (TARE) with the purpose of facilitating controlled fractional microsphere administration for a more flexible and image-guided TARE procedure.

**Methods:**

A Controlled Administration Device (CAD) was developed using MR-conditional materials. The CAD contains a rotating syringe to keep the microspheres in suspension during administration. Different rotational speeds were tested ex vivo to optimise the homogeneity of microsphere fractions administered from the device. The technical performance, accuracy, and safety was validated in three patients in a clinical TARE setting by administering a standard clinical dose in 5 fractions (identifier: NCT05183776). MRI-based dosimetry was used to validate the homogeneity of the given fractions in vivo, and serious adverse device event ((S)A(D)E) reporting was performed to assess safety of the CAD.

**Results:**

A rotational speed of 30 rpm resulted in the most homogeneous microsphere fractions with a relative mean deviation of 1.1% (range: -9.1-8.0%). The first and last fraction showed the largest deviation with a mean of -26% (std. 16%) and 7% (std. 13%). respectively. In the three patient cases the homogeneity of the microsphere fractions was confirmed given that MRI-based dosimetry showed near linear increase of mean absorbed target liver dose over the given fractions with R^2^ values of 0.98, 0.97 and 0.99. No (S)A(D)E’s could be contributed to the use of the CAD.

**Conclusions:**

The newly developed CAD facilitates safe and accurate fractional microsphere administration during TARE, and can be used for multiple applications in the current and future workflows of TARE.

**Supplementary Information:**

The online version contains supplementary material available at 10.1186/s40658-024-00692-6.

## Background

Transarterial radioembolisation (TARE) is a locoregional treatment modality used for treatment of liver tumours of various origins [1–3]. During TARE, microspheres containing a beta-emitting isotope are injected in the hepatic artery. Due to the preferential blood flow of the hepatic artery, the microspheres mostly end up in the tumours, where they lodge in the microvasculature due to their size (~ 30 μm) to locally irradiate the tissue. Currently, three types of microspheres are commercially available, containing the isotope yttrium-90 (^90^Y) or holmium-166 (^166^Ho). Both isotopes have a therapeutic property due to their beta emittance and can be imaged using the nuclear imaging modalities PET and bremsstrahlung SPECT (^90^Y), and SPECT (^166^Ho) [4, 5]. Furthermore, the microspheres containing ^166^Ho are paramagnetic and therefore they can be visualised using MRI. During the last decade, quantitative imaging and dosimetry of ^166^Ho microspheres using MRI has been established and can now be used in a standardised way to evaluate the patient treatment [6–9].

The clinical value of using dosimetry in TARE has become apparent during the last years, mainly due to the ancillary study of the SARAH trial and the DOSISPHERE study [10–12]. The SARAH study investigated retrospective dosimetry of ^90^Y resin microspheres for advanced stage HCC patients and concluded that an absorbed tumour dose of > 100 Gy corresponded to an increased overall survival compared to an absorbed tumour dose of < 100 Gy (median 14.1 months and 6.1 months respectively) [11]. The DOSISPHERE-01 study had similar outcomes in a prospective study using ^90^Y glass microspheres in HCC patients, as a targeted absorbed tumour dose > 205 Gy corresponded to an increased overall survival compared to the standard 120 Gy to the perfused lobe group (26.6 months and 7.1 months respectively) [10]. These findings clearly indicate the importance of treatment personalisation and the use of dosimetry for TARE. Treatment personalisation could however be taken a step further.

In current clinical practice, the MR imaging properties of ^166^Ho microspheres are only used for post-treatment evaluation, as is done with conventional nuclear imaging techniques in TARE. However, ^166^Ho MRI can also provide quantitative information during an image-guided TARE procedure. The first steps towards an image-guided TARE procedure were studied by Roosen et al., who concluded that administering microspheres while the patient is positioned in the MR scanner is feasible and that an image-guided procedure might improve the patient’s treatment [13, 14]. If ^166^Ho microspheres (^166^Ho-MS) are administered in fractions and imaging is performed in between the given fractions, the distribution of the ^166^Ho-MS can be evaluated at different stages of the procedure. Intraprocedural changes in characteristics such as the tumour-to-normal ratio (T/N ratio) can then be accounted for during treatment. Catheter positions and administered activity can be optimised during treatment, which could lead to an even more personalised approach for TARE.

In current clinical practice ^166^Ho-MS are flushed out of the delivered V-Vial and into the catheter by injecting saline solution into the V-Vial at a rate of 5 mL/min, as per the instructions for use (IFU). This is alternated by flushing the tubing to administer the ^166^Ho-MS to the hepatic blood flow and to inspect the catheter position and the presence of back flow using a contrast agent. The design of the administration system leads to an uncontrolled administration profile as the concentration of administered ^166^Ho-MS varies with each flush [15]. Implementation of an image-guided TARE procedure would require full control of the administered ^166^Ho-MS of each fraction, using a device that allows for a linear release profile of the microspheres into the microcatheter. This enables the user to make intraprocedural decisions on the ^166^Ho-MS to be administered and the location on which they need to be administered.

The primary objective of this work was to design and validate a novel medical device for fractional, linear microsphere administration during TARE. The technical performance, accuracy and safety of the novel device was optimised ex vivo. Finally, the safety and accuracy of the optimised device was validated in a small patient cohort.

## Methods

### Device design

The purpose of the novel controlled administration device (CAD) is to administer freely chosen fractions of known amounts of ^166^Ho-MS. To realize this, a homogeneous ^166^Ho microsphere suspension must be made, maintained and administered. However, microspheres settle when suspended due to the higher density of the ^166^Ho microspheres (1.41 g/cm^3^) compared to the administration fluid (1.00 g/cm^3^), hampering a homogenous microsphere suspension and thus controlled administration of microspheres. The main requirement of the device therefore was to create a setup enabling a continuous homogeneous microsphere suspension from which small fractions can be administered. Additional requirements for the device included: (1) accurate and manual syringe controlled administration (2), administration in an MR environment (3), flushing of the administration lines and catheter (4), loading of the device from a V-Vial with rubber septum and (5) appropriate radiation safety of the device (comparable to the current administration box). Based on those aspects a device was developed to enable controlled administration in an MRI-guided setting (patent# *NL2030571B1*).

**Fig. 1:**
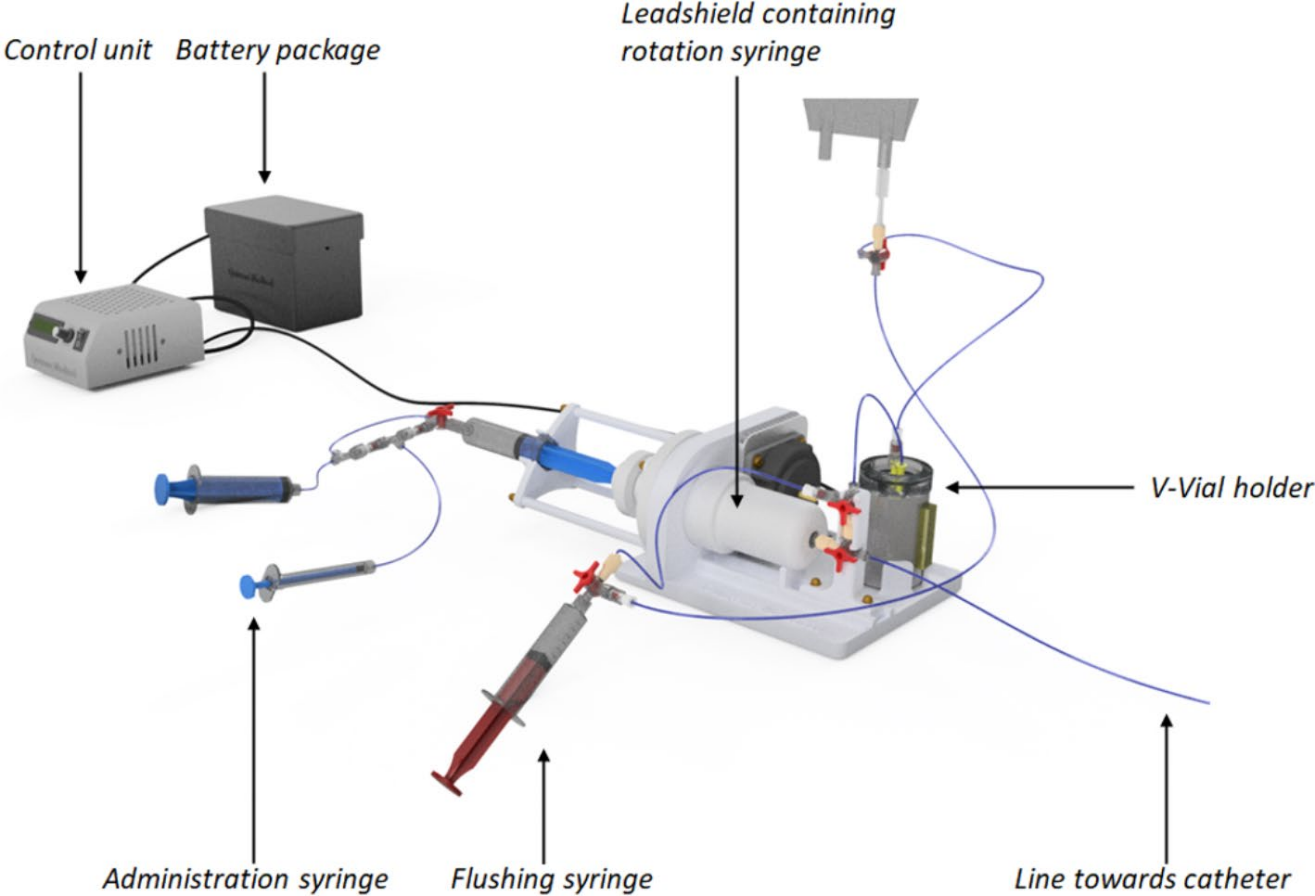
Schematic overview of the Controlled Administration Device (CAD). A syringe enclosed by 10 mm of lead contains the ^166^Ho-MS suspension. This syringe rotates using a piezo motor, as a result of which the microspheres stay in suspension. The hydraulic actuation system consisting of 3 administration syringes (2 × 20 mL, and 1 mL, on the left) is used to control the rotating syringe. The first 20 mL syringe is positioned back to back with the rotating syringe in an empty position. The second 20 mL and the 1 mL syringes can be used to fill the rotating syringe in the system. The small syringe is also used to control the administration of the microsphere suspension in small amounts through the catheter. Flushing can be performed using the 10 mL syringe illustrated on the bottom

The design consists of a mechanism that allows to rotate a syringe to bring the ^166^Ho microspheres in suspension (Fig. 1). If a continuous homogeneous microsphere solution is formed, then the activity in an administered fraction can be described by the relation:

1 $$\:{A}_{target}={V}_{administration}\cdot\:{C}_{suspension}$$

With $\:{A}_{target}$ the target activity to be administered (MBq), $\:{V}_{administration}$ the volume to be administered (mL) and $\:{C}_{suspension}$ the activity concentration in the suspension volume (MBq/mL).

The rotating syringe is connected to a hydraulic actuation system that contains a 1 mL syringe to control the administration volume. The administration system is composed of non-magnetic parts to be MR-safe whereas the MR-conditional control unit and battery pack are connected via a 5 m electric cable for placement outside the 0.50 mT field of the MRI. A flushing syringe is connected to the administration disposables for flushing the catheter and the lines towards the catheter. Radiation shielding of 10 mm lead was applied.

### Ex vivo validation

#### Homogenisation

To investigate the homogeneity of the suspension, administration tests with rotational speeds of 20 rpm (*n* = 5), 30 rpm (*n* = 5) and 40 rpm (*n* = 5) were performed with non-radioactive holmium-165 (^165^Ho) microspheres. During the tests the 20 mL syringe of the CAD was fully loaded with 600 ± 25 mg of ^165^Ho microspheres in 2 mL 0.1% Pluronic buffer (Quirem Medical B.V., Deventer, The Netherlands) and the remaining volume was filled with 0.9% NaCl. To determine the homogeneity of the suspension and the stability of the administered fractions, the total volume in the rotating syringe was manually administered in 10 fractions of 2 mL via a microcatheter (Progreat™ 2.7 Fr, 150 cm, Terumo Corporation, Tokyo, Japan). Each administered fraction was followed by a 10 mL 0.9% NaCl flush. The fractions were separately collected in 50 mL centrifuge tubes (Griener Bio-one International GmbH, Kremsmünster, Austria). After the ^165^Ho microspheres settled in each centrifuge tube, the supernatant was removed and the remaining microspheres were dried in a Binder VD53 vacuum oven (Binder GmbH, Tuttlingen, Germany) at a temperature of 45 °C and under a pressure of 0.05 MPa for a period of 24 h. Subsequently, the weight of each vial was measured using an Ohaus EX225D analytical balance (OHAUS Corporation, Parsippany, USA).

The optimal rotational speed was subsequently applied in a test with ^166^Ho microspheres representing a clinical situation. During these tests (*n* = 4), 3 mL V-Vials loaded with ^166^Ho microspheres in 2 mL Pluronic buffer were transferred to the rotating syringe by flushing 0.9% NaCl through the V-Vial until the 20 mL rotating syringe in the device was filled. A similar administration profile as described before was applied during which 2 mL suspension fractions and 10 mL flushes were administered via a microcatheter. Using a dose calibrator (VDC-603, Comecer, Joure, The Netherlands), the administered fractions as well as the remaining activity in the 3 mL V-Vials were measured to determine the transfer efficiency from the V-Vial to the rotating syringe in the administration system.

#### MR-compatibility

To facilitate administering dose in an MR-environment, the device was designed to be MR-safe and MR-conditional. The CAD characteristics in an MRI environment were tested in practise by placing the device directly next to the bore of a preclinical 7T MR-scanner (Bruker ClinScan, Billerica, Massachusetts, USA) and a clinical 3T MR-scanner (MAGNETOM Skyra, Siemens Healthineers, Erlangen, Germany) while magnetic induced displacements were assessed visually. The clinical MR-scanner was also used to determine the effects on the RF field by measuring the RF field with an operational device (i.e. running motor) and non-operational device.

### In vivo validation

#### Study design and participants

In addition to ex vivo testing, the CAD was evaluated in a clinical study to ensure safety, usability and accuracy of the device in an image-guided TARE setting. This was done in a single-centre pilot clinical study, conducted at the Radboud University Medical Center (Nijmegen, The Netherlands). Eligible participants were ≥ 18 years and had a diagnosis of either HCC or liver-dominant mCRC, considered eligible for TARE by the tumour board. Minimal life expectancy of the patients was 12 weeks with a WHO performance status of 0–2. Major exclusion criteria were ineligibility for MR imaging; systemic, surgical or radiation therapy within four weeks before TARE; serum bilirubin > 26 µmol/L; glomerular filtration rate (MDRD) < 35 mL/min; leukocytes < 4.0 × 10^9^/L and platelet count < 60 × 10^9^/L; and alanine aminotransferase (ALT), aspartate aminotransferase (AST) or alkaline phosphatase (AP) > 5 × the upper limit of normal. Study protocol details were published on clinicaltrials.gov (identifier: NCT05183776).

All patients provided written informed consent, and the ethical review board committee approved the protocol (METC Oost-Nederland, ref. NL78931.091.21). The study was performed in accordance with the Declaration of Helsinki and Good Clinical Practice.

#### Study procedures

All patients underwent standard pre-treatment imaging, pre-treatment work-up and dose planning, but only MR-safe materials were used for the pre-treatment work-up to ensure usability of these materials during treatment. During the pre-treatment work-up, catheter positions were determined using visceral angiography, and QuiremScout™ was administered to determine tumour uptake of the microspheres and possible shunting. Activity planning was based on the T/N ratio on SPECT/CT, the treated volume and the liver function. The maximally prescribed activity was in accordance with a whole liver mean absorbed dose of 60 Gy in all cases as described in the HEPAR trial [16] and as is advised in the QuiremSpheres IFU.

The treatment procedure took place one to two weeks after the pre-treatment work-up at the Medical Innovation and Technology expert Center (MITeC) in an MRI-guided TARE setting as was done before in the EMERITUS study [13]. At the MITeC, a cone-beam CT (CBCT; Artis Pheno, Siemens Healthineers, Erlangen, Germany) and a 3T MRI (MAGNETOM Skyra, Siemens Healthineers, Erlangen, Germany) are located in adjacent rooms. After a pre-treatment MRI scan, the patient was transferred to the CBCT for catheter placement. The patient was transferred back to the MRI once the catheter was in position to administer the microspheres from the CAD. In case of multiple catheter positions, only one catheter position was treated according to the study protocol, while the other positions were treated according to standard clinical practise using the administration box.

The use of a medical device in an MRI environment comes with several additional safety risks. To limit the safety burden extensive warnings were placed in the research protocol and the device components were labelled with MR-safe and MR-conditional labels for use during the clinical study. During the placement of the catheter at the CBCT, the CAD was assembled and prepared for administration. The preparation included assembling the disposable tubing system, priming the system with saline solution to remove any air from the tubing and loading the microspheres from the V-Vial into the rotating syringe using saline. The CAD was then turned on to homogenise the microsphere suspension in the syringe. This was done at a rotational speed of 70 rpm for two minutes to enforce movement of the settled microspheres, after which the system was set to 30 rpm to homogenise the suspension and perform the administration. The total activity in the syringe was administered in 5 fractions of 4 mL, and after each administration, an MRI was made to quantify the dose distribution.

#### Data-analysis

The data acquired during the clinical study was used to validate the CAD in an in vivo clinically relevant setting. Technical performance in vivo was validated by the duration of the injection, defined as the time between two MRI acquisitions. Furthermore, technical performance was also defined by the loss of activity in the system, which was determined by measuring the remaining activity in the V-vial, disposables and catheter after the treatment was finished using the dose calibrator. The accuracy of the administrations was defined as the linearity of the dose increase based on absorbed dose on the treated liver volume. The treated liver absorbed dose was calculated for each given fraction based on the MRI-scans in a research version of Qsuite 2.1™ (Quirem Medical B.V. – a Terumo company, Deventer, The Netherlands). Lastly, safety was assessed by monitoring serious adverse events (SAEs) and serious adverse device events (SADEs) (CTCAE version 4.03) from the inclusion date until three months after treatment. Safety assessment was performed at the time of the procedure, 1 week after treatment, 6 weeks after treatment and 3 months after treatment.

## Results

### Ex vivo testing

**Fig. 2:**
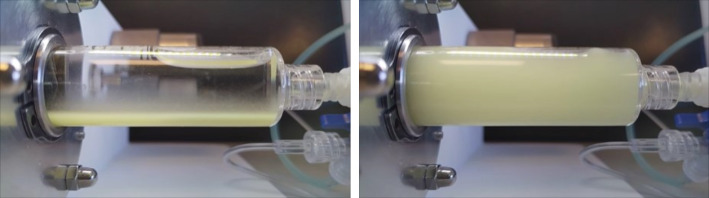
Settled holmium-165 microspheres in 0.9% NaCL loaded in syringe before homogenisation (left) and after homogenisation at rotational speed of 70 rpm for 2 min (right)

**Fig. 3:**
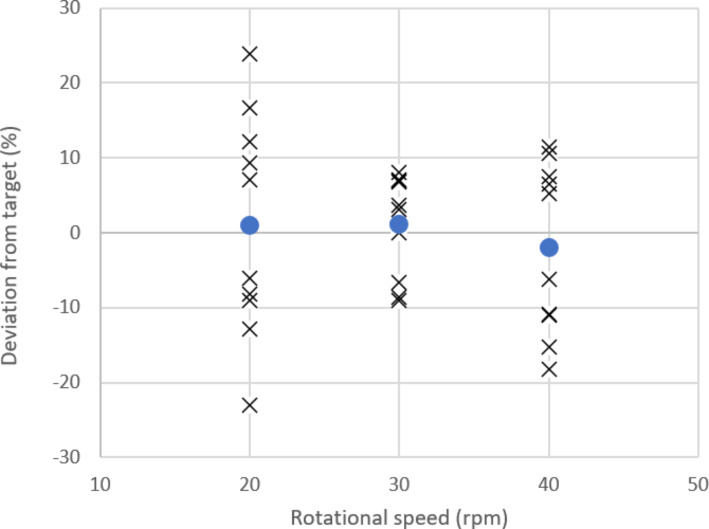
Mean deviation percentage of administered fractions from the target value at rotational speeds of the rotating syringe at 20, 30 and 40 rpm. For each rotational speed, 10 administration tests were performed. The mean deviation is represented by the dot, the deviation per test is represented by the crosses. A rotational speed of 30 rpm shows the smallest deviations from the targeted administration amount

Continuous rotation of the syringe containing ^165^Ho microspheres led to visual homogenisation (Fig. 2). Fractional administration of the microsphere suspension at a rotational speed of 20, 30 and 40 rpm resulted in a mean deviation from the targeted amount of microspheres of 1.0% (-23.0-23.8%), 1.1% (-9.1-8.0%) and − 2.0% (-18.2-11.5%) as can be seen in Fig. 3.

**Fig. 4:**
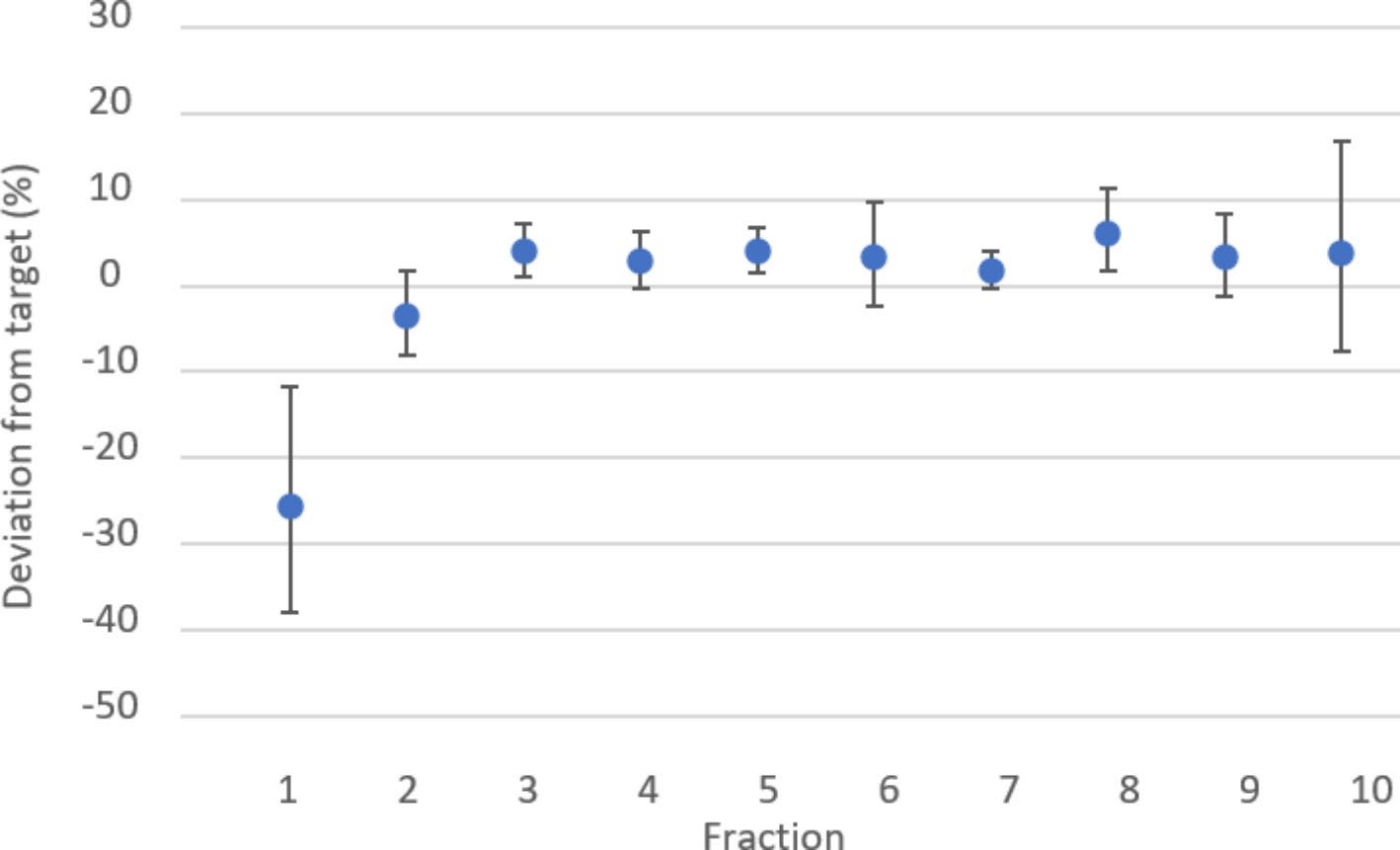
The mean deviation of the expected administered fractions. Each fraction was 2 mL and would therefore represent 10% of the total syringe volume, the Y-axis represents the percentual deviation from the expected administered fraction. On the X-axis the 10 different fractions are shown in chronological order. The error bars represent the standard deviation for each fraction. The first and last administered fraction show the highest variability

As the fractions were considered most homogeneous at a rotational speed of 30 rpm, this was used for the follow-up experiments with varying amounts of ^166^Ho microspheres (exact amounts can be found in Table A1 in the supplementary material). The distribution of the microspheres over 10 given fractions for a therapeutic dose of ^166^Ho microspheres at 30 rpm are presented in Fig. 4. For all 4 tests, the deviations from the expected administered activity for fraction 2 to 10 were found to be within ± 10% of the average of all these fractions. The first fraction showed a larger deviation from the expected administered activity with a mean of -26% (std. 16%). The last fraction was within the 10% limit, but showed a high standard deviation (mean deviation of 7% with std. 13%). The measurements of the left activity values in the V-Vials showed losses of on average 1.35% (std. 1.73%).

### In vivo testing

#### Patient and treatment characteristics

Five patients were found eligible and were included in the clinical study cohort. One patient was excluded after the pre-treatment work-up due to significant lung shunting based on ^166^Ho scout. The four remaining patients received ^166^Ho-MS TARE at the MITeC. Patient characteristics can be found in the supplementary material. During the treatment session, the therapy of a single patient was relocated to the CBCT because of catheter instability and continued catheter displacement, delaying the treatment. Consequently, no MRI data was obtained to study the validity of the device for this patient. However, safety analysis was performed as usual as microsphere administration was performed using the CAD. In the other three patients, the treatment was successfully performed according to the study protocol. Thus, both safety and validity of the CAD could be assessed in these three patients.

#### Device validation

The median duration of one fractionated administration including imaging and setup was 7 min (5–18 min), including image acquisition of 3 min. Therefore the median duration of a single administration including flushing was 4 min (2–15 min). The activity loss due to activity that remained in the tubing, vials and catheters was 9.0%, 10.5% and 8.1% for the three treated patients. Most of the loss was due to the disposables of the CAD (tubing, 3-way stopcocks and syringe), which contained 80.2%, 86.93% and 71.0% of the total waste of activity (Table 1).

**Table 1:** The activity to be administered according to the study procedure and the actually administered activity due to loss of activity in the vial, disposable components and the catheter. The majority of the activity loss was due to the disposable components of the CAD

	Patient 1	Patient 2	Patient 3
Vial activity (MBq)	1822.8	2900.8	4735.1
Waste (MBq, % of total) Vial Catheter Disposable components	163.7 (9.0%) 23.6 MBq (1.3%) 8.8 MBq (0.5%) 131.3 MBq (7.2%)	304.9 (10.5%) 39.1 MBq (1.3%) 0.8 MBq (< 0,1%) 265.0 MBq (9.1%)	383.3 (8.1%) 99.4 MBq (2.1%) 11.8 MBq (0.2%) 272.2 MBq (5.7%)
Administered activity (MBq)	1659.1 (91.0%)	2595.9 (89.5%)	4351.8 (91.9%)

**Fig. 5:**
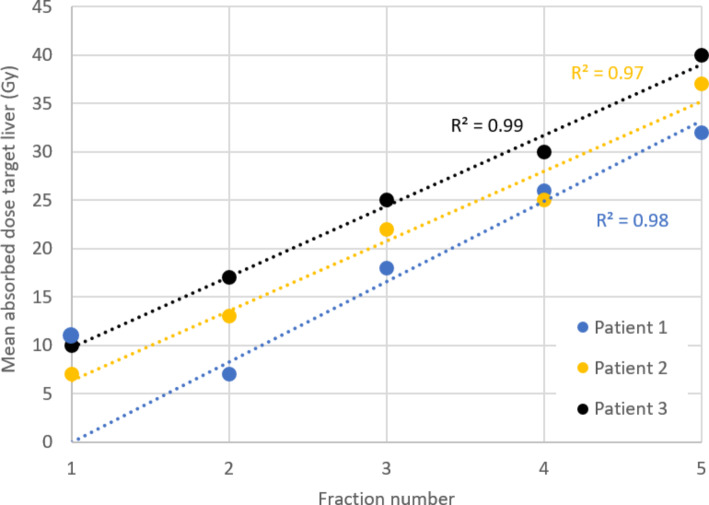
The dose in the targeted liver volume after each fraction administered with the CAD. The MRI-based absorbed dose after each fraction for the three patients that received the treatment with the CAD at the MRI are represented in dots. The dashed lines show the linear relationship of the dose over time. The R^2^ of the fit of the data to the linear relationship is reported in the colour respective to the specific patient (0.98; 0.97; 0.99 respectively)

**Fig. 6:**
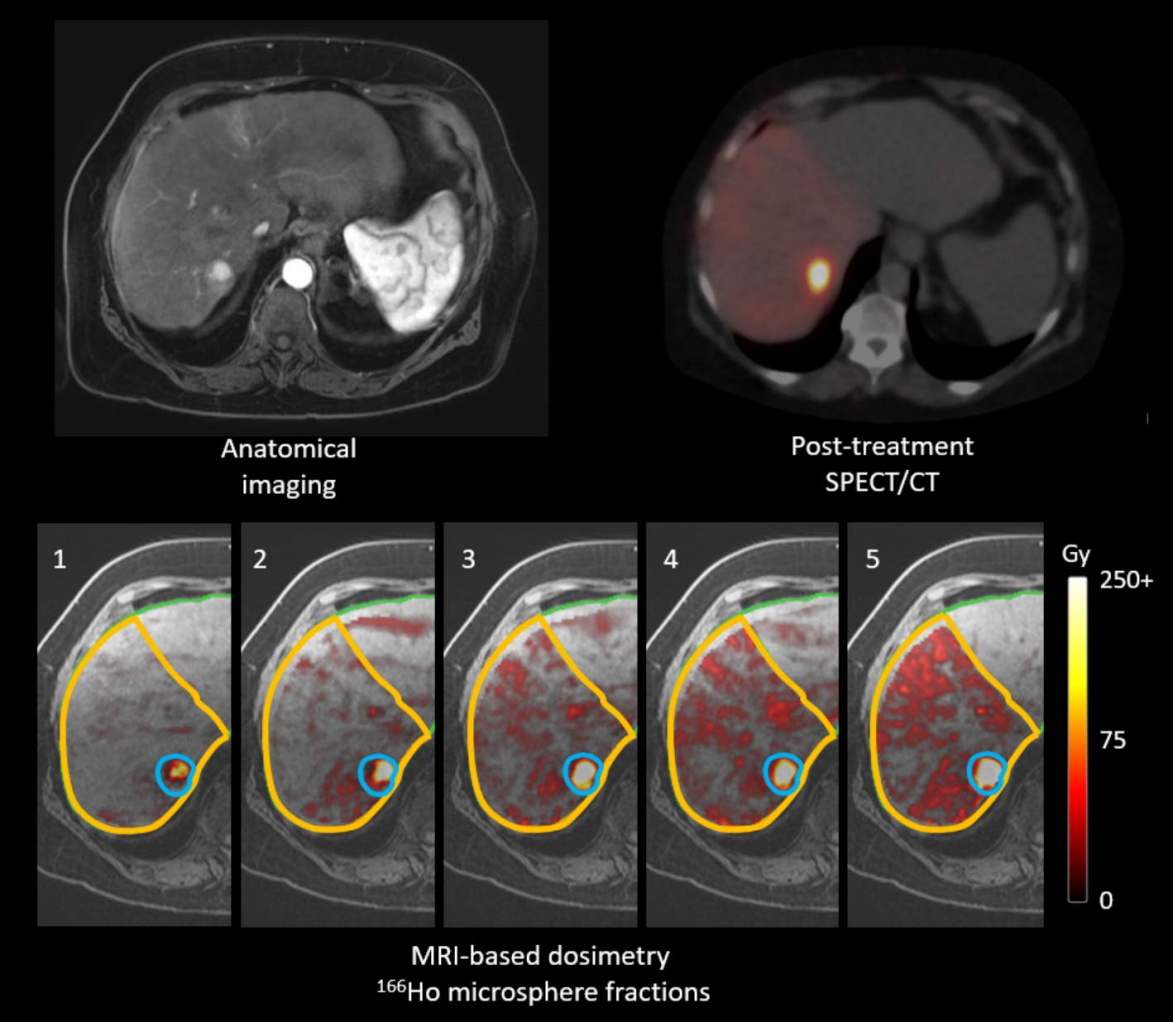
Study patient 2 with pre-treatment contrast-enhanced MRI, showing a lesion in segment 7. Post treatment ^166^Ho SPECT/CT acquired five days after treatment shows high uptake in the lesion and low homogeneous uptake in the treated non-tumoural liver. MRI-based dose maps after each given microsphere fraction using the CAD. The treated liver part is delineated, within the treated liver part the tumour is delineated. Each fraction represents an increase of 20% of the planned dose administration. The tumour dose increases with each given fraction, as well as the non-tumoural liver tissue

The MRI quantification showed an absorbed dose increase with each fraction in both tumour and non-tumoural liver (Fig. 5). The R^2^ of the linear relation of the recovered dose with MRI over the five fractions was 0.98, 0.97 and 0.99 respectively per patient. For the first patient the recovered dose of the first fraction was not included in the R^2^ calculation, as the receiver coils of the MRI were partially switched off, which led to inaccurate dose recovery on MRI. The near 1.00 R^2^ values indicate accurate linear increase of administered activity by the CAD, which can also be appreciated in a patient example in Fig. 6.

#### Device safety

The four patients treated with ^166^Ho-MS using the CAD were all included in the safety analysis. No (S)AEs could be attributed to use of the CAD. Four serious adverse events (SAEs) occurred, in two patients. The first patient received an additional TARE treatment 3 months after the study treatment, 1 month after this additional treatment the patient was hospitalised because of grade 3 and eventually grade 4 REILD [17]. The second patient was diagnosed with REILD grade 3 based on clinical (ascites, deterioration of liver enzymes) and radiological evaluation (lack of tumour progression). Other AEs were mild (grade 1 or 2) and frequently reported side effects of the therapy such as fatigue and nausea. These symptoms did not need any intervention. A list of all (S)AEs can be found in the supplementary data.

## Discussion

Recent literature has highlighted the potential clinical advantages of enhancing treatment personalisation in TARE, primarily through personalised pre-treatment dosimetry, aiming to attain an adequate tumour dose [10–12]. While these recent approaches have demonstrated the potential of personalised TARE, achieving personalised dose administration while maintaining control of the quantity of administered microspheres during treatment has not been attainable yet. The current administration devices all have the same working mechanism and do not facilitate controlled administration of freely chosen fractions from a single vial. In this article, we have developed and validated an innovative device for controlled administration of ^166^Ho-MS during TARE.

First, the administration device was optimised and validated in an ex vivo setting representative of the clinical workflow. Three different rotational speeds were tested to achieve a homogeneous microsphere suspension, required for controlled ^166^Ho-MS administration during TARE. A rotational speed of 30 rpm resulted in a homogeneous microsphere suspension, yielding the most uniform fractions (deviations within 10%). This optimal rotational speed can be explained by the fact that at a lower rotational speed, gravity results in microspheres settling at the bottom of the syringe, whereas at a higher rotational speed the centrifugal forces on the microspheres will cause microspheres settling to the wall of the syringe, resulting in an inhomogeneous suspension. The first fraction of microspheres consistently showed less outflow of activity (deviation of -26% as opposed to the desired ± 10%), and the last fraction more outflow compared to the other fractions (deviation of 7% with a high standard deviation). This could be explained by the priming of the system during injection of the first fraction, as microspheres will first precipitate in dead volumes of three-way stopcocks as was previously shown by Drescher et al. [15]. The deviation of the first fraction can be minimized by flushing thoroughly, and by anticipating on the precipitation by administering a slightly bigger fraction. During the last fraction, it is hypothesized that slightly more microspheres are administered because the syringe is being pushed to the empty position. In practise this will not always occur, since in the majority of cases the syringe will not be emptied completely during a personalised treatment and excess activity will remain in the syringe. Overall, it was considered that the uncertainties of the administered activity over the different fractions were within an acceptable frame for clinical implementation.

Second, the design and ex vivo results were further validated in a small patient population. As MRI-based dosimetry resulted in a clear linear increase of the target liver dose as more microspheres were injected, we conclude that the accuracy of the device in vivo is in line with the ex vivo tests. Minor deviations from the linear dose increase of the MRI-based data could be induced by the device but could also be induced by artefacts from the lungs and bowels, and other uncertainties of the MRI-based dosimetry [7, 13]. These deviations are too small to have an influence on clinical decision making and were therefore accepted. During clinical evaluation, no device-related (S)A(D)Es were found. All AEs were well-known side-effects of the TARE treatment, such as fatigue, nausea and abdominal pain and could therefore not be attributed to the use of the CAD [16, 18, 19]. The SAEs were remarkable, as REILD occurred in two out of four patients. REILD is a set of clinical signs that come from liver toxicity due to a high dose on the hepatocytes [17]. In the two described cases, classic clinical signs as ascites and deterioration of liver enzymes without radiological tumour progression were observed. One REILD case likely resulted from TARE re-treatment, while the other occurred in a whole liver treatment where no non-tumoural liver tissue could be spared. Therefore, despite the relatively high REILD incidence, the SAEs could not be attributed to the use of the novel device.

The administrations were performed according to the MRI-guided TARE protocol in three of the four patients with a median administration duration of 7 min, including an MRI acquisition of 3 min. This is slightly faster than during the EMERITUS study, during which the mean administration time (including imaging) was 10 min [13]. The duration is comparable to the administration of microspheres of a complete V-Vial in current clinical practice which is around 8 min [15]. It should be noted that a new system must be prepared for each catheter position for the conventional administration system, while the CAD system can contain the activity for all positions. Since the preparation time for the conventional and the CAD system is comparable, the use of the CAD system significantly saves time in TARE procedures requiring multiple catheter positions. Above all, the dose distribution is obtained with high resolution MRI during treatment within the 7 min of administration time. In current clinical practise, a SPECT is needed, which has a lower spatial resolution, longer acquisition time, and requires an additional hospital visit due to the high count rate directly after treatment [20]. Additionally, it is challenging to perform quantitative measurements using holmium SPECT [20], while extensive methods to perform quantitative holmium MRI have been developed during the last decade [6, 7, 9]. Therefore, quantitative MRI might fill the gap of quantitative imaging for ^166^Ho-MS to improve holmium TARE personalisation.

Although the CAD has primarily been designed for image-guided TARE, it could provide significant value in terms of flexibility for conventional TARE as well. Firstly, the CAD only requires a single vial for a complete treatment, as several user-defined fractions of microspheres can be administered in a linear fashion across different catheter positions. Additionally, no separate administration box has to be prepared for each new catheter position. Secondly, in case of a scout procedure a standard scout dose could be ordered, and the amount of catheter positions and the amount of scout activity to be administered at each catheter position can be determined during the procedure based on the perfused volume. A caveat is the low number of microspheres in a scout dose compared to a treatment dose, as a result of which a relatively large number of microspheres could be lost in priming the system. Lastly, having the complete dosing in a single system facilitates swift adjustments in dosing, creating more flexibility. This could for example simplify a one-day treatment. Currently patient specific vials have to be ordered, which means dose planning has to be performed days in advance [21]. If a general dosing vial with at least the maximum needed activity could be ordered for use with the CAD, the treating physician could determine the dosing after the work-up in the morning and administer only the necessary fraction of activity. Using the CAD for multiple catheter positions would mean that the catheters need to be disconnected, which could lead to a contamination. This situation needs to be investigated during further research.

There are more clinical situations during which TARE could benefit from controlled microsphere administration. For instance during radiation segmentectomy, it can be favourable to continue microsphere administration until stasis of arterial blood flow occurs, such as in transarterial chemoembolisation. The occurrence of stasis in TARE is difficult to predict, but limited tumour vascularity and small treated volume sizes are hypothesized to be causes [22, 23]. The CAD allows for more careful injection of microspheres (in smaller, controlled fractions). The more linear administration pattern of the CAD compared to the administration boxes [15] could potentially be an advantage in preventing early stasis due to bolus injections, leading to hypothetically increased absorbed tumour doses. This has been explored by Miller et al., with a dual syringe approach, leading to a more linear administration profile compared to the conventionally used boxes. The more linear administration profile led to more (^90^Y) microspheres reaching the distal tumour vasculature [24]. It can therefore be hypothesized that a lower microsphere concentration in the arterioles due to linear administration will result in a deeper penetration of the tumour vasculature because obstruction of the flow in the arterioles is prevented. Therefore, the overall lower and stable microsphere concentration in the CAD compared to the extreme high concentration during the first administration through the conventional system might result in a benefit for the dose distribution.

The use of the CAD also indicates room for improvement. During this study, only a single catheter position for each patient was treated with the device. Multiple catheter positions necessitates the disconnection and reconnection of catheters. Replacing and disconnecting catheters can lead to ^166^Ho contamination and needs further research and standardisation to limit risks during the procedure. In case of incorporation in the scout workflow, further investigation in the loss of activity within the system should be performed. The loss of microspheres in the current system is around 10%, and for both the scout and therapeutic dose a minimal loss of activity would be preferable in order to relate the administered activity to the imaging before activity measurements and to limit radiation exposure. Given the limited shielding around the tubing and three-way stopcocks, radiation safety can be further improved by reducing losses in those parts. Therefore, an optimisation in the disposable system which would be limiting the amount of connecting points would improve the system and thereby improve treatment using the CAD.

Overall, the newly designed controlled administration system has several advantages compared to the currently used systems, specifically in facilitating personalised image-guided TARE, but also by increasing flexibility in the treatment workflow and potentially in the scout workflow.

## Conclusion

The newly developed controlled administration device allows for precise and safe administration of freely chosen ^166^Ho microsphere fractions. This paves the way towards more personalised and image-guided TARE, but also increases flexibility and shows potential for current applications of ^166^Ho TARE.

## Electronic supplementary material

Below is the link to the electronic supplementary material.

Supplementary Material 1

## Data Availability

The datasets and analyses that support the findings of this study are available from the corresponding author upon reasonable request.
