# Supplementary material for: Development and validation of an innovative administration system to facilitate controlled holmium-166 microsphere administration during TARE
Source: EJNMMI Phys. 2024 Oct 16;11:87. doi: 10.1186/s40658-024-00692-6 (PMC11484995; doi:10.1186/s40658-024-00692-6)
Supplement: Supplementary file 1 — Supplementary Material 1 [file 40658_2024_692_MOESM1_ESM.docx]

**Supplementary material**

Table A1 - Used amount of microspheres and related (specific) activities.

|  | Amount of microspheres [mg] | Total activity [MBq] | Specific activity [MBq/mg] |
| --- | --- | --- | --- |
| Vial 1 | 779.53 | 219.00 | 0.27 |
| Vial 2 | 990.73 | 326.00 | 0.33 |
| Vial 3 | 598.53 | 52.20 | 0.08 |
| Vial 4 | 1008.32 | 70.50 | 0.07 |

Table A2: Characteristics of the patients that got treatment using the CAD.

|  | Patient 0 (excluded) | Patient 1 | Patient 2 | Patient 3 |
| --- | --- | --- | --- | --- |
| Age | 76 | 71 | 63 | 80 |
| Sex | Male | Female | Female | Male |
| Primary malignancy | Hepatocellular carcinoma | Hepatocellular carcinoma | Hepatocellular carcinoma | Colorectal carcinoma |
| Prior treatment | ablation | NA | NA | 6 cycles CAPOX-B |
| WHO performance | 0 | 1-2 | 2 | 0 |
| Extrahepatic disease | NA | NA | NA | NA |
| Lab results   - Creatinine - Bilirubine - ALT - AST - Alkaline phosphatase | 91 umol/L  17 umol/L  63 mmol/L  60 mmol/L  186 U/L | 55 umol/L  31 umol/L  8 mmol/L  43 mmol/L  236 U/L | 64 umol/L  14 umol/L  41 mmol/L  47 mmol/L  132 U/L | 100 umol/L  7 umol/L  26 mmol/L  37 mmol/L  108 U/L |
| Total liver mass (kg) | 1853 | 1021 | 1258 | 1554 |
| Targeted liver part | Whole liver | Right hemiliver, segment 3 | Right hemiliver | Whole liver |
| Catheter positions | 2 | 2 | 1 | 2 |
| Planned total liver dose | 60 Gy | 60 Gy | 37 Gy | 60 Gy |

Table A3: (serious) adverse events that occurred from study inclusion until 3 months post TARE. No events could be attributed to the use of a new administration device

| (S)AE | Grade 1 | Grade 2 | Grade 3 | Grade 4 | SAE |
| --- | --- | --- | --- | --- | --- |
| Nausea | 3 | 1 |  |  |  |
| Fatigue | 1 | 1 |  |  |  |
| Stomach pain |  | 1 | 1 |  |  |
| Headache | 1^a^ |  |  |  |  |
| Inguinal hematoma |  |  | 1 |  |  |
| Diarrhea | 1 |  |  |  |  |
| Back pain |  | 2 |  |  |  |
| Gastric haemorrhage^b^ |  |  |  |  | 1 |
| REILD^c^ |  |  |  |  | 2 |

^a^ Headache associated with periods of fatigue, ^b^ Need for hospitalisation, ^c^ REILD
